# Cellular Differences in the Cochlea of CBA and B6 Mice May Underlie Their Difference in Susceptibility to Hearing Loss

**DOI:** 10.3389/fncel.2019.00060

**Published:** 2019-02-27

**Authors:** Huihui Liu, Gen Li, Jiawen Lu, Yun-Ge Gao, Lei Song, Geng-Lin Li, Hao Wu

**Affiliations:** ^1^Department of Otolaryngology-Head and Neck Surgery, Shanghai Ninth People's Hospital, Shanghai Jiao Tong University School of Medicine, Shanghai, China; ^2^Ear Institute, Shanghai Jiao Tong University School of Medicine, Shanghai, China; ^3^Shanghai Key Laboratory of Translational Medicine on Ear and Nose Diseases, Shanghai, China

**Keywords:** hearing loss, hair cell, spiral ganglion neuron, ribbon synapse, calcium current, exocytosis

## Abstract

Hearing is an extremely delicate sense that is particularly vulnerable to insults from environment, including drugs and noise. Unsurprisingly, mice of different genetic backgrounds show different susceptibility to hearing loss. In particular, CBA/CaJ (CBA) mice maintain relatively stable hearing over age while C57BL/6J (B6) mice show a steady decline of hearing, making them a popular model for early onset hearing loss. To reveal possible underlying mechanisms, we examined cellular differences in the cochlea of these two mouse strains. Although the ABR threshold and Wave I latency are comparable between them, B6 mice have a smaller Wave I amplitude. This difference is probably due to fewer spiral ganglion neurons found in B6 mice, as the number of ribbon synapses per inner hair cell (IHC) is comparable between the two mouse strains. Next, we compared the outer hair cell (OHC) function and we found OHCs from B6 mice are larger in size but the prestin density is similar among them, consistent with the finding that they share similar hearing thresholds. Lastly, we examined the IHC function and we found IHCs from B6 mice have a larger Ca^2+^ current, release more synaptic vesicles and recycle synaptic vesicles more quickly. Taken together, our results suggest that excessive exocytosis from IHCs in B6 mice may raise the probability of glutamate toxicity in ribbon synapses, which could accumulate over time and eventually lead to early onset hearing loss.

## Introduction

The dysfunction and/or loss of sensorineural cells in cochlea often result in hearing impairment, which is a major social and health problem worldwide (Fujimoto et al., 2017). Sensorineural hearing loss involves in mixed pathology including loss of hair cells and/or spiral ganglion neurons (SGNs), atrophy of stria vascularis, decline of endolymphatic potential and cochlear anatomical alterations (Schuknecht and Gacek, 1993). Hair cells, including inner hair cells (IHCs) and outer hair cells (OHCs) in cochlea, play an essential role in converting sound vibrations into neural signals. SGNs are the first order of spike-generating neurons in the auditory pathway, and they connect hair cells to neurons in cochlear nuclei in the brainstem. However, both of them are prone to be damaged by multiple and complex underlying etiologies, such as aging, acoustic exposure, ototoxic drugs, genetic disorders, etc. (Mammano and Bortolozzi, 2018). Exploring possible mechanisms of sensorineural cell dysfunction will expand our understanding of neural and molecular basis of this sensory deficit.

Animal models for studying hearing loss have been utilized over half a century and mice are now the widely used species for hearing research (Ohlemiller, 2006). Among them, CBA/CaJ (CBA) and C57BL/6J (B6) mice are two of the most useful animal models for investigating hearing loss. B6 mice are highly susceptible to acoustic overstimulation while CBA mice are fairly resistant to noise trauma (Davis et al., 2001). Previous studies had found that B6 mice have a rapid hearing loss, reaching severe levels by about 1 year of age. In contrast, CBA mice have excellent cochlear sensitivity and a slow progressive hearing loss over age, so that they maintain sensitive hearing well into old ages (Jimenez et al., 1999). B6 mice carry a *cadherin23* (*Cdh2*3) mutation, also known as *Ahl* (Noben-Trauth et al., 2003). *Cdh23* encodes cadherin 23, which is critically important for hair cell development. Specifically, cadherin 23 is required for proper maintenance of hair cell structures such as stereociliary tip links (Siemens et al., 2004; Kazmierczak et al., 2007), kinocilial and transient lateral links (Lagziel et al., 2005; Michel et al., 2005). Mutations in *Cdh23* cause kinocilium displacement and splayed stereocilia during early hair cell differentiation (Di Palma et al., 2001). Another locus, *Ahl3*, has been shown to cause hearing loss and is also identified in B6 (Nemoto et al., 2004). Besides gene expression differences between the two mouse strains, significant morphological differences in the cochlea are also found at the age of 6 months, including efferent nerve endings under OHCs and SGNs (Park et al., 2010, 2011).

However, structural and functional differences in early ages could affect the development and progress of hearing loss and there has been a lack of side-by-side comparison of cellular functions in the cochlea of these two mouse strains, especially before hearing loss starts. To fill this knowledge gap, we used juvenile animals with normal hearing to explore cellular differences in the cochlea of these two mouse strains, aiming to identify candidate mechanisms of early onset hearing loss.

## Materials and Methods

### Auditory Brainstem Responses (ABRs)

Male CBA/CaJ (CBA) and C57BL/6J (B6) mice of 4 weeks old (SIPPR-BK Laboratory Animal Ltd., Shanghai, China) were used throughout this study. All animal handling was complied with the Guide for the Care and Use of Laboratory Animals (8th edition), published by the National Institutes of Health (NIH, USA) and approved by the University Committee of Laboratory Animals of Shanghai Jiao Tong University.

For ABR recording, animals were anesthetized with chloral hydrate (480 mg/Kg i.p.) and the body temperature was maintained near 37°C with a heating blanket. ABRs were recorded via three sub dermal needle electrodes, placed at the vertex of skull, the mastoid area of test ear and the shoulder of opposite side. Short tone pips of 3 ms that consist 1 ms rise and fall cosine ramp were delivered at 20 Hz rate through a computer-generated acoustic stimulation system (Tucker-Davis Technologies). For each frequency and each sound pressure level (SPL), 400 responses were collected and averaged in BioSigRZ (Tucker-Davis Technologies). All ABR recordings were conducted by the same experimenter. Hearing thresholds were defined as the lowest SPL that can evoke appropriate ABR responses, in 5 dB steps descending from 90 dB SPL (Song et al., 2006). The amplitude and latency of ABR Wave I was measured manually offline.

### Whole-Cell Patch-Clamp Recording

Patch-clamp recordings were performed in IHCs and OHCs in the apical turn of basilar membrane through an EPC10/2 amplifier (HEKA Electronics, Lambrecht Pfalz, Germany), driven by Patchmaster (HEKA Electronics). Recording pipettes were pulled from borosilicate glass capillaries (World Precision Instruments) and coated with dental wax. The pipettes had a typical resistance of about 6 MΩ when filled with pipette solution containing (in mM) 135 Cs-methane sulfonate, 10 CsCl, 10 HEPES, 10 TEA-Cl, 1 EGTA, 3 Mg-ATP, and 0.5 Na-GTP (290 mOsm, pH 7.20). Extracellular solution contained (in mM) 110 NaCl, 2.8 KCl, 25 TEA-Cl, 5 CaCl_2_, 1 MgCl_2_, 2 Na-pyruvate, 5.6 D-glucose, and 10 HEPES (300 mOsm, pH 7.40). All patch-clamp experiments were carried out at room temperature and the liquid junction potential was corrected offline.

Conductance-voltage relationships were calculated from Ca^2+^ current responses to ramp stimulation from −90 mV to +50 mV and fitted to the Boltzmann equation:

$$\text{G}\left(\text{V}\right)=\frac{{\text{G}}_{\max}}{1+{\text{e}}^{\frac{{\text{V}}_{\text{half}}-\text{V}}{{\text{k}}_{\text{slope}}}}}$$

where V is the command membrane potential, G_max_ is the maximum conductance, V_half_ is the half-activation voltage, k_slope_ is the slope factor that defines steepness of voltage dependence in current activation.

For IHCs, whole-cell membrane capacitance measurements were performed with the lock-in feature and “Sine + DC” method in Patchmaster (HEKA). Briefly, sine waves of 1 kHz and 70 mV (peak-to-peak) were superposed on the holding potential, and the resulting current responses were used to determine whole-cell capacitance (C_m_). The net increase of C_m_ before and after stimulation (ΔC_m_) was used to assess exocytosis from IHCs.

For OHCs, whole-cell capacitance was measured with an Axon 200B amplifier (Molecular Devices, Sunnyvale, CA) and a two-sine voltage command (10 to 20 mV, with a primary frequency of 390.6 Hz and harmonic of 781.2 Hz), superimposed on to a voltage ramp (Santos-Sacchi and Song, 2014). The extracellular solution contained (in mM) 100 NaCl, 20 TEA-Cl, 20 CsCl, 2 CoCl_2_, 1 MgCl_2_, 1 CaCl_2_, and 10 HEPES (300 mOsm, pH 7.40). The pipette solution contained (in mM) 140 CsCl, 2 MgCl_2_, 10 HEPES, and 10 EGTA (300 mOsm, pH 7.20). Two-state Boltzmann function was used to fit linear and non-linear capacitance data:

$${\text{C}}_{\text{m}}(\text{V})={\text{Q}}_{\max}\times \frac{\text{ze}}{\text{kT}}\times \frac{\text{b}}{{\left(1+\text{b}\right)}^{2}}+{\text{C}}_{\text{lin}};\text{b}={\text{e}}^{\frac{-\text{ze}(\text{V}-{\text{V}}_{\text{half}})}{\text{kT}}}$$

Where, Q_max_ is the maximum non-linear charge moved, V_half_ is the half-maximum voltage, z is the valence, and C_lin_ is the linear membrane capacitance, i.e., the resting membrane capacitance.

### Whole-Mount Cochlear Staining

Cochleae were acutely dissected after animals were anesthetized deeply and sacrificed. For fluorescence staining, the apical turn of basilar membranes was excised and fixed with 4% paraformaldehyde (PFA) for 2 h, permeabilized with 5% Triton X-100 for 30 min, and blocked in 5% BSA for 60 min. The tissue was then incubated overnight at 4°C with two primary antibodies, mouse anti-CtBP2 (BD Biosciences, catalog # 612044, 1:200 dilution) and mouse anti-GluR2 (Millipore, catalog # MAB397, 1:100 dilution). Next day, after three washes in PBS, the tissue was incubated with two secondary antibodies, Alexa Fluor 568-conjugated goat anti-mouse IgG1 (Invitrogen, catalog # A-21124, 1:200 dilution) and Alexa Fluor 647-conjugated IgG2 (Invitrogen, catalog # A-21241, 1:200 dilution). Fluorescence images were acquired on a Zeiss confocal microscope (LSM 510 META).

For histological staining, cochleae were fixed in 4% PFA overnight at 4°C, decalcified in PBS with 10% EDTA for 7 days at 4°C and dehydrated in 30% sucrose. Cochleae were then embedded in OCT, frozen at −20°C, and sectioned parallel to the modiolus at 5 μm per section (ThermoFisherScientific Inc., Waltham, MA). The slides were dried, washed with PBS for three times, blocked in 0.1% Triton X-100 with 5% BSA in PBS for 1 h at room temperature and incubated overnight at 4°C with the primary antibody mouse anti-TUJ1 (Covance, catalog # MMS-435P, 1:500 dilution). HRP-conjugated rat anti-mouseIgG2a secondary antibody (Invitrogen, catalog#04-6220, 1:500 dilution) was used to visualize the primary antibodies and the slides were stained with DAB. Images were captured with a digital camera, and the density of spiral ganglion neurons (SGNs) was calculated for the apical, medial and basal turn of cochleae, by dividing the number of SGNs with the area surveyed.

### Data Analysis and Statistical Tests

Data were analyzed in Igor Pro (WaveMetrics, USA) with home-made macros and statistical tests were performed in Prism (GraphPad, USA) with built-in functions. Depending on the nature of data set, statistical significance was assessed with unpaired Student's *t*-test, Mann-Whitney test or two-way ANOVA followed by Bonferroni *post-hoc* test. Data are presented as Mean ± SD in text and as Mean ± SEM in figures, and the level of significance was set to *p* < 0.05. In figures, N.S. means *p* > 0.05, ^*^means *p* < 0.05, ^**^ means *p* < 0.01, and ^***^ means *p* < 0.001.

## Results

### Hearing Performance

**Figure 1:**
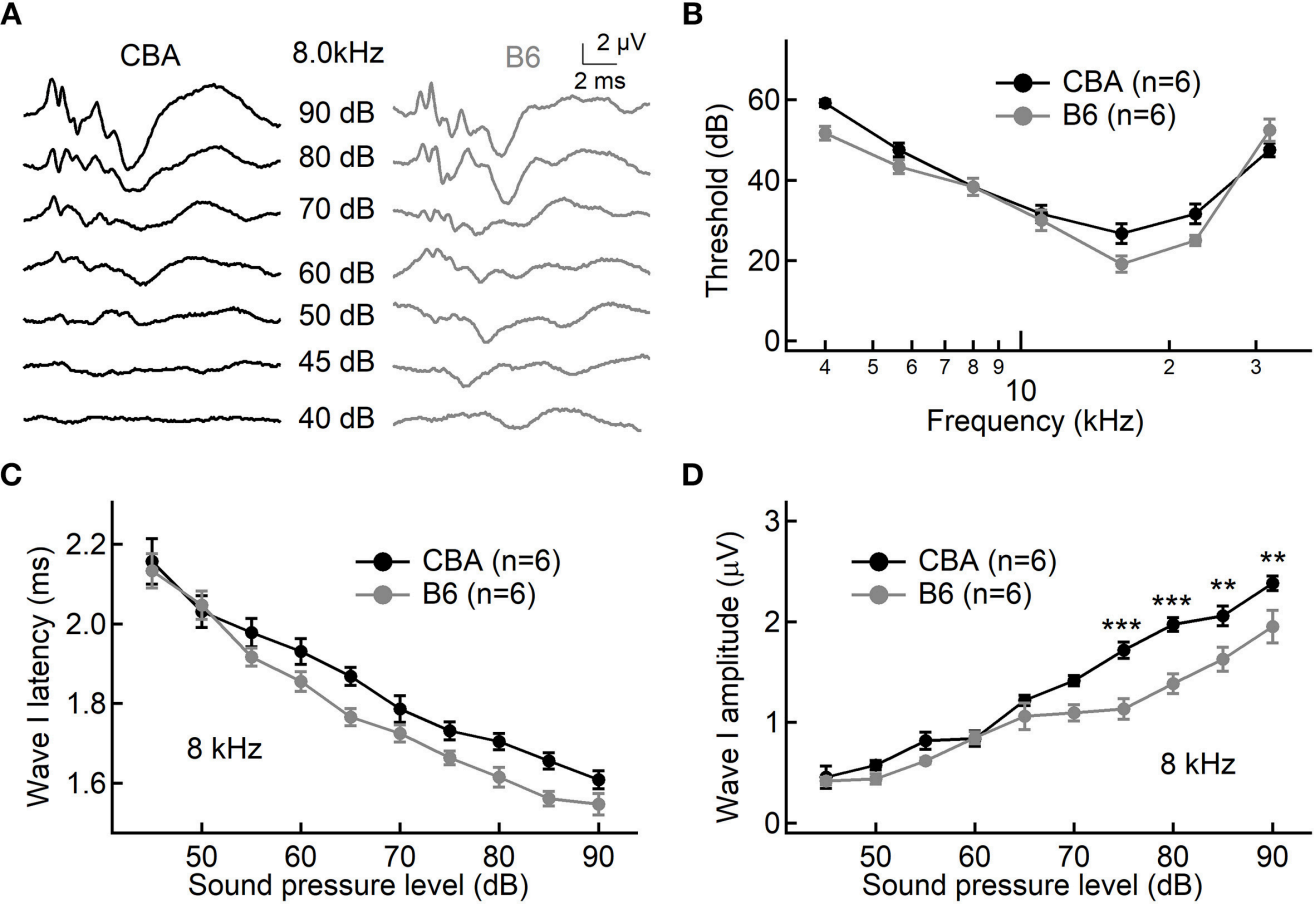
Hearing performance of juvenile CBA and B6 mice. **(A)** Representative auditory brainstem responses (ABRs) recorded from two mice, one from each strain. **(B,C)** Across all frequencies tested, no significant differences were found in either the ABR threshold **(B)** or ABR Wave I latency **(C)** between the two mouse strains. **(D)** As the sound pressure level (SPL) went beyond 70 dB, the ABR Wave I amplitude from B6 mice became significantly smaller than that of CBA mice. For both mouse strains, pure tone pips of 8 kHz with an increasing SPL were presented to induce ABRs. For all figures, data are depicted as Mean ± SEM, ^**^ means *p* < 0.01, and ^***^ means *p* < 0.001.

To examine differences of hearing performance between CBA and B6 mice, we presented short tone burst to animals under anesthesia and recorded auditory brainstem responses (ABRs). This is a non-invasive way to assess hearing performance, and the first wave, i.e., Wave I, represents summated activity of responding auditory afferent fibers (Figure 1A). Consistent with previous studies (Frisina et al., 2007; Ohlemiller et al., 2016; Hickox et al., 2017), we found no significant difference between the two mouse strains in either the ABR threshold or Wave I latency (*n* = 6 for both mouse strains, two-way ANOVA, *p* > 0.05, Figures 1B,C).

Next, we examined ABR Wave I amplitude at 8 kHz, a frequency located in the apical turn. We chose to focus on the apical turn for this entire study because this is the only region in adult cochlea where hearing epithelium can be excised with sufficient tissue integrity for patch-clamp analysis on inner hair cells (IHCs), an approach we would like to take for later experiments. Although there is no significant difference for low sound pressure levels (SPLs, 45–70 dB), Wave I amplitude is significantly smaller for B6 mice at high SPLs starting from 75 dB (1.72 ± 0.20 vs. 1.13 ± 0.25 μV, *n* = 6 for both mouse strains, two-way ANOVA, *p* < 0.001). This difference in the Wave I amplitude for louder sounds is not unique to the apical turn, as we observed similar difference for both medial and basal turn (16 and 32 kHz, data not shown).

### Inner Hair Cell (IHC) Function

**Figure 2:**
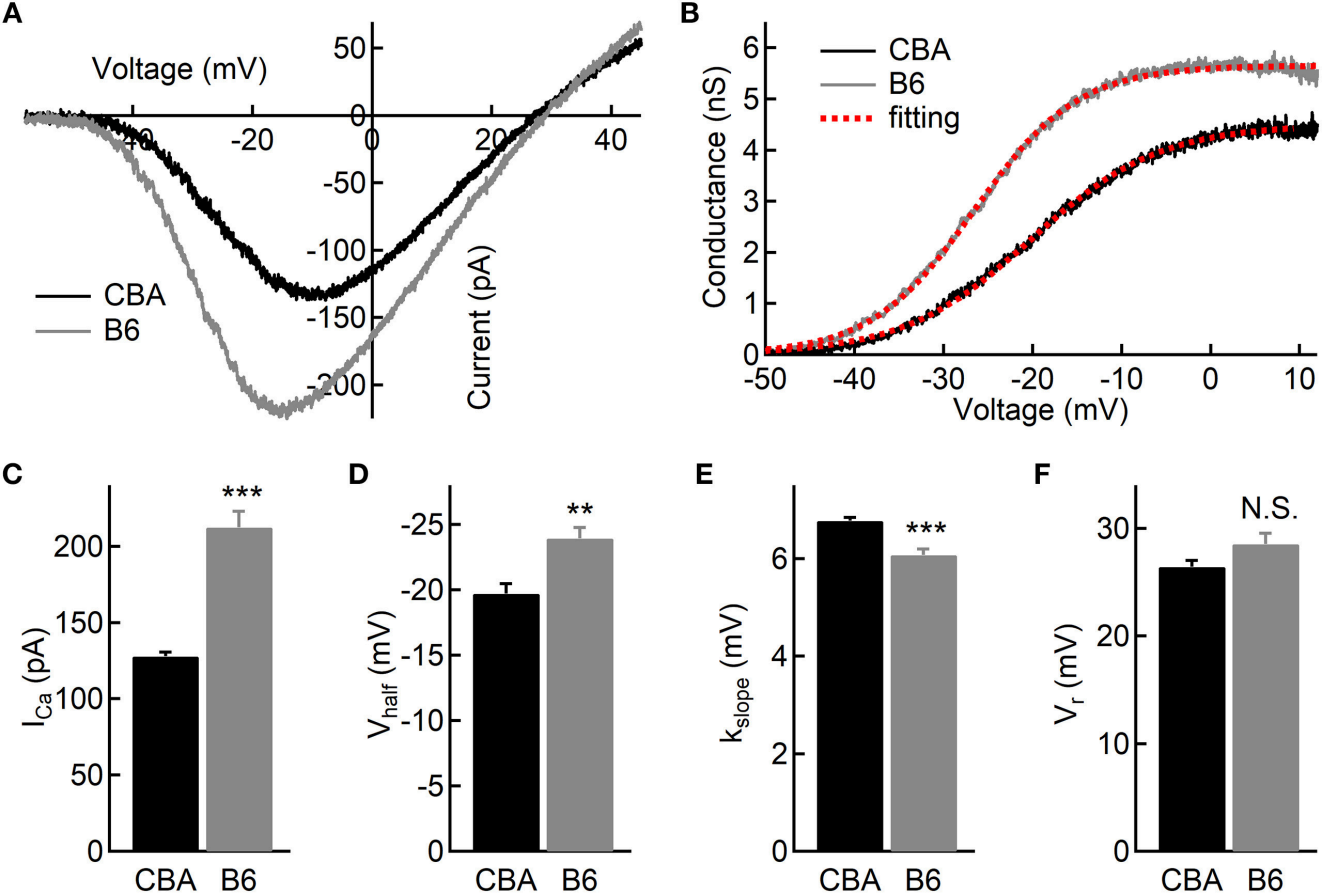
Ca^2+^ current in inner hair cells (IHCs) of CBA and B6 mice. **(A)** Representative Ca^2+^ currents recorded from two IHCs, one from each mouse strain, induced by a voltage ramp from −90 to +50 mV under voltage-clamp. **(B)** Based on the Ca^2+^ current shown in **(A)**, the conductance was calculated point-by-point and plotted against the membrane potential applied. The dashed lines in red depict Boltzmann fitting. **(C–E)** The Ca^2+^ current in IHCs from B6 mice has a larger peak amplitude (I_Ca_, **C**), a more negative half-activation voltage (V_half_, **D**) and a steeper voltage dependency (k_slope_, **E**). **(F)** No statistical significance was found in the reversal potential of the Ca^2+^ current (V_r_) between the two mouse strains. N.S. means *p* > 0.05, ^**^ means *p* < 0.01 and ^***^ means *p* < 0.001.

To examine functional differences in IHCs between the two mouse strains, we conducted whole-cell patch-clamp recording in IHCs from the apical turn. We first applied ramp stimulation and recorded the Ca^2+^ current (I_Ca_, Figure 2A). We found that the peak amplitude of I_Ca_ is significantly larger in IHCs from B6 mice (−128 ± 12.3 vs. −212 ± 37.0 pA, *n* = 17 and 12 cells; Mann-Whitney *U-*test, *p* < 0.001; Figure 2C). As expected, we found no significant different in the Ca^2+^ reversal potential between the two mouse strains (26.4 ± 2.57 vs. 28.5 ± 3.58 mV, *n* = 17 and 12; unpaired Student's *t*-test, *p* > 0.05; Figure 2F). Next, we converted I_Ca_ to conductance point-by-point and fitted conductance-voltage relationship to the Boltzmann function (Figure 2B), yielding the half-activation voltage (V_half_) and the slope of activation (k_slope_). We found that I_Ca_ in B6 mice has a more negative V_half_ (−19.7 ± 3.17 vs. −23.9 ± 2.95 mV, *n* = 17 and 12; unpaired Student's *t*-test, *p* < 0.01; Figure 2D) and a steeper activation slope (6.77 ± 0.28 vs. 6.07 ± 0.44 mV, *n* = 17 and 12; unpaired Student's *t*-test, *p* < 0.001; Figure 2E), suggesting that Ca^2+^ channels in B6 IHCs could bring more Ca^2+^ influx into the cell at physiological conditions.

**Figure 3:**
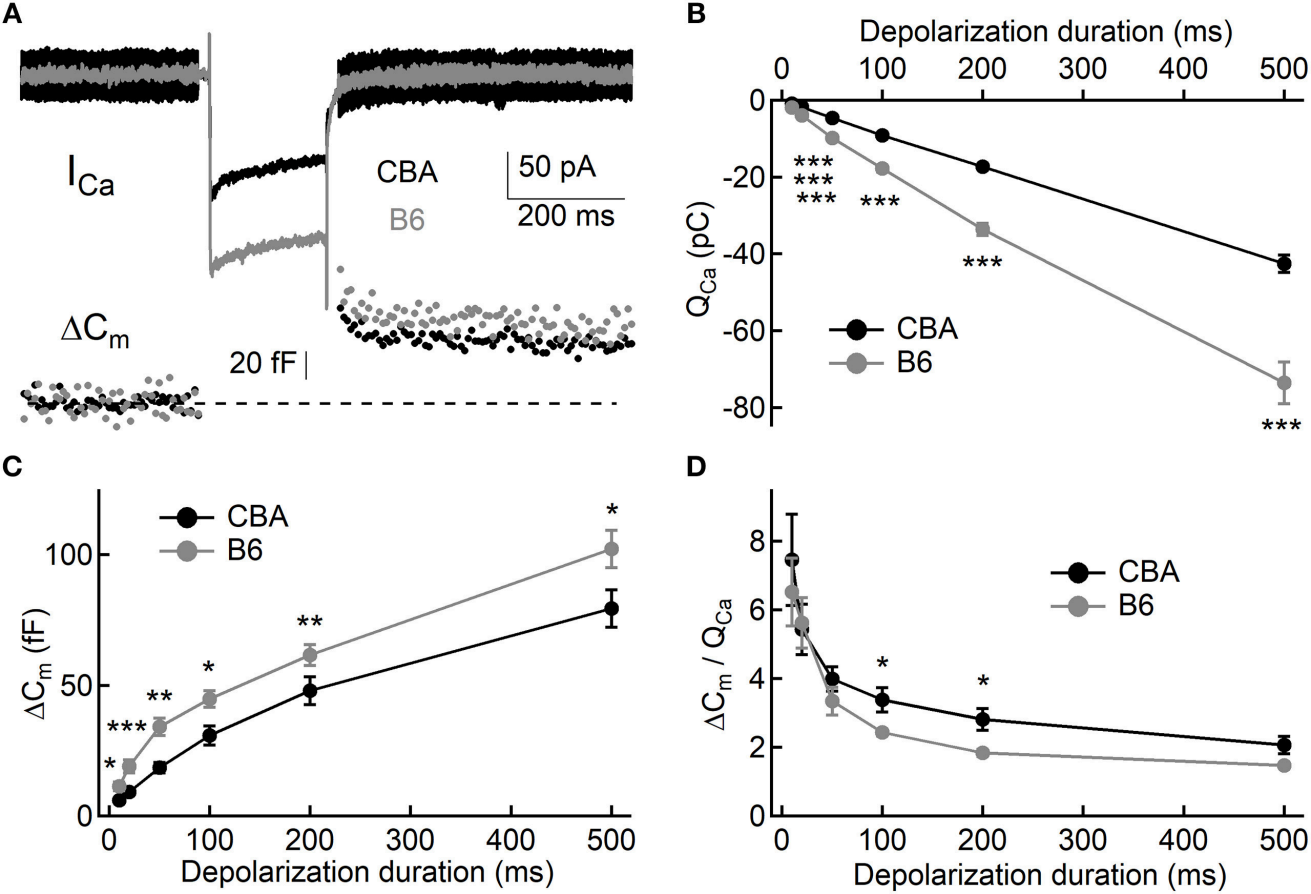
Exocytosis from IHCs. **(A)** Representative Ca^2+^ currents and whole-cell capacitance measurements in two IHCs, one from each mouse strain. Small sine waves were superposed on the holding potential before and after a step depolarization. The step depolarization induced a Ca^2+^ current (I_Ca_) and triggered exocytosis, which can be quantified with capacitance increase (ΔC_m_). **(B,C)** Both Ca^2+^ influx (Q_Ca_, **B**) and exocytosis (ΔC_m_, **C**) are significantly larger in IHCs from B6 mice. **(D)** Ca^2+^ efficiency in triggering exocytosis, i.e., the ratio of ΔC_m_/Q_Ca_, is significantly lower in IHCs from B6 mice. ^*^ means *p* < 0.05, ^**^ means *p* < 0.01 and ^***^ means *p* < 0.001.

**Figure 4:**
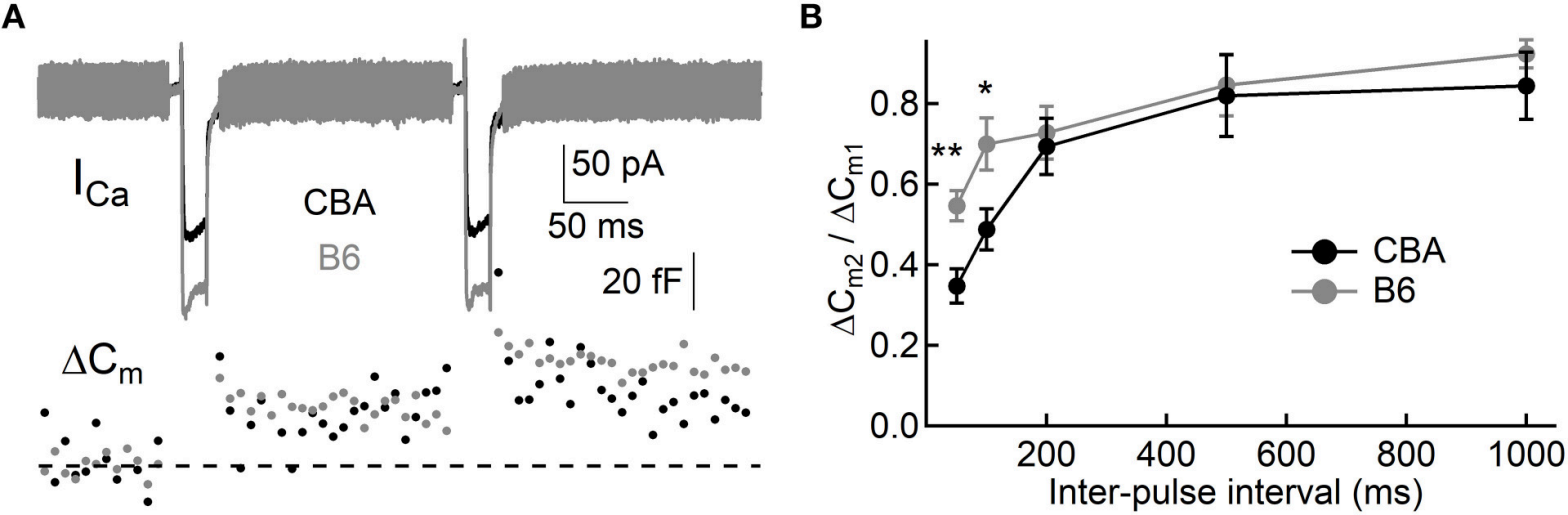
Synaptic vesicle replenishment in IHCs. **(A)** Representative current responses of two IHCs to double pulse stimulation, one from each mouse strain. Both pulses (20 ms) induced notable I_Ca_ and ΔC_m_, and the ratio of ΔC_m2_/ΔC_m1_ can be calculated and used to quantify synaptic vesicle replenishment. **(B)** Synaptic vesicle replenishment was significantly faster in IHCs from B6 mice. ^*^ means *p* < 0.05 and ^**^ means *p* < 0.01.

To assess exocytosis in IHCs, we applied step stimulation and conducted whole-cell capacitance measurement (Neher and Marty, 1982) to determine membrane area increase (ΔC_m_, Figure 3A). We varied stimulation duration from 10 to 500 ms, and we found more Ca^2+^ influx (Q_Ca_) in IHCs from B6 mice (see Table 1 and Figure 3B), consistent with the aforementioned finding that I_Ca_ in B6IHCs has a larger peak amplitude. Additionally, we found IHCs from B6 mice released more synaptic vesicles for both short and long stimulation (see Table 1 and Figure 3C), suggesting that they have a large readily releasable pool of synaptic vesicles and that they replenish synaptic vesicles more efficiently. Although they release more synaptic vesicles, the Ca^2+^ efficiency of triggering exocytosis, assessed with the ratio of ΔC_m_/Q_Ca_, is less efficient for stimulation of both 100 and 200 ms (Table 1 and Figure 3D). To directly examine synaptic vesicle replenishment, we applied double-pulse stimulation with different intervals and built recovery curves of exocytosis for IHCs from both mouse strains (Figure 4). We found that indeed exocytosis recovered quicker for IHCs from B6 mice. For an interval of 50 ms, ΔC_m_ in CBA mice recovered to 0.35 ± 0.16 (*n* = 14) while ΔC_m_ in B6 mice recovered to 0.55 ± 0.13 (*n* = 13, unpaired Student's *t*-test, *p* < 0.01). Similar result was observed for an interval of 100 ms (0.49 ± 0.20 vs. 0.70 ± 0.22, *n* = 15 and 12, unpaired Student's *t*-test, *p* < 0.05), reinforcing the idea that synaptic vesicle replenishment is more efficient in IHCs of B6 mice.

**Table 1:** Summary of Δ*C*_m_, Q_Ca_, and ΔC_m_/*Q*_Ca_ in IHCs.

		**10 ms**	**20 ms**	**50 ms**	**100 ms**	**200 ms**	**500 ms**
ΔC_m_	CBA (fF)	6.03 ± 2.06 (*n* = 11)	9.20 ± 3.95 (*n* = 17)	18.5 ± 7.75 (*n* = 15)	30.8 ± 12.7 (*n* = 12)	43.6 ± 11.4 (*n* = 11)	79.4 ± 21.3 (*n* = 9)
	B6 (fF)	13.2 ± 7.44 (*n* = 10)	22.0 ± 9.80 (*n* = 11)	32.4 ± 8.86 (*n* = 17)	43.7 ± 9.43 (*n* = 10)	61.3 ± 12.4 (*n* = 7)	107.2 ± 22.5 (*n* = 8)
*P-value*		Mann-Whitney tests (*p* = 0.015)	Unpaired student's *t*-test (*p* = 0.0004)	Unpaired student's *t*-test (*p* = 0.0013)	Unpaired student's *t*-test (*p* = 0.015)	Unpaired student's *t*-test (*p* = 0.007)	Unpaired student's *t*-test (*p* = 0.02)
Q_ca_	CBA (pC)	−0.91 ± 0.21 (*n* = 11)	−1.81 ± 0.36 (*n* = 17)	−4.65 ± 0.83 (*n* = 15)	−9.14 ± 1.55 (*n* = 12)	−17.3 ± 2.62 (*n* = 11)	−42.6 ± 6.95 (*n* = 9)
*P-value*	B6 (pC)	−1.96 ± 0.38 (*n* = 10) Unpaired student's *t*-test (*p* < 0.0001)	−3.97 ± 0.86 (*n* = 11) Unpaired student's *t*-test (*p* < 0.0001)	−9.84 ± 1.23 (*n* = 17) Unpaired student's *t*-test (*p* < 0.0001)	17.7 ± 2.70 (*n* = 10) Unpaired student's *t*-test (*p* < 0.0001)	−33.6 ± 4.08 (*n* = 7) Unpaired student's *t*-test (*p* < 0.0001)	−73.5 ± 15.3 (*n* = 8) Unpaired student's *t*-test (*p* < 0.0001)
ΔC_m_/*Q*_Ca_ *P-value*	CBA (fF/pC) B6 (fF/pC)	7.46 ± 4.39 (*n* = 11) 6.51 ± 3.13 (*n* = 10) Unpaired student's *t*-test (*p* = 0.58)	5.43 ± 3.04 (*n* = 17) 5.62 ± 2.41 (*n* = 11) Unpaired student's *t*-test (*p* = 0.86)	3.99 ± 1.36 (*n* = 15) 3.34 ± 1.08 (*n* = 17) Unpaired student's *t*-test (*p* = 0.94)	3.38 ± 1.22 (*n* = 12) 2.43 ± 0.33 (*n* = 10) Unpaired student's *t*-test (*p* = 0.03)	2.58 ± 0.81 (*n* = 11) 1.83 ± 0.28 (*n* = 7) Mann-Whitney tests (*p* = 0.046)	1.95 ± 0.76 (*n* = 9) 1.47 ± 0.18 (*n* = 8) Mann-Whitney tests (*p* = 0.16)

*Summary of ΔC_m_, Q_Ca_, and ΔC_m_/Q_Ca_ from patch-clamp recordings in IHCs (Figures 3B,C). Data are presented mean ± SD; n = number of IHCs; statistical tests and p-values are presented for each dataset*.

### Outer Hair Cell (OHC) Function

**Figure 5:**
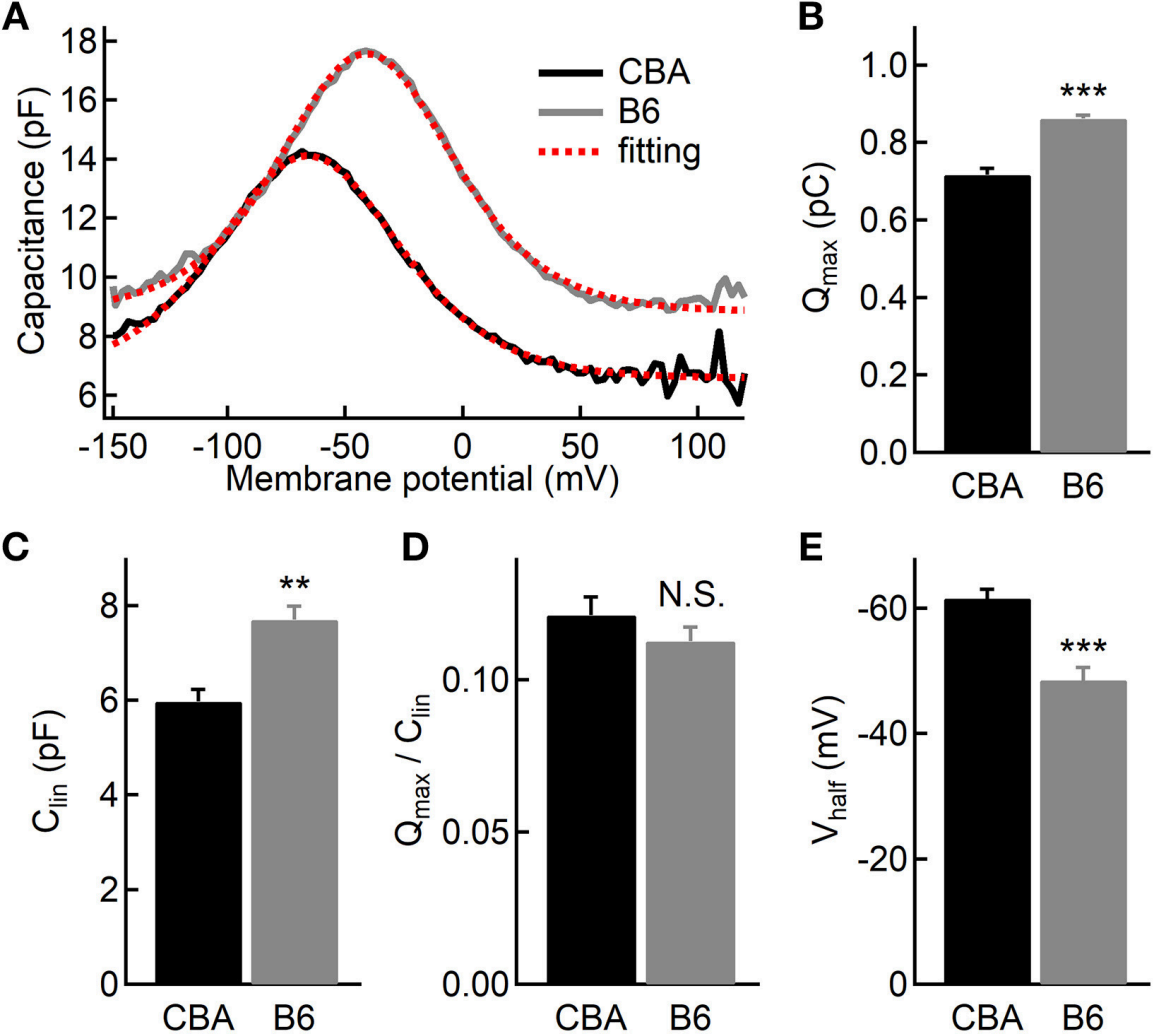
Electromotility of outer hair cells (OHCs). **(A)** Capacitance recordings from two OHCs in response to a voltage ramp. The dashed lines in red depict two-state Boltzmann fitting (see the Materials and Methods). **(B–D)** Both non-linear **(B)** and linear **(C)** capacitances are significantly larger in OHCs from B6 mice, but the ratio of the two parameters is comparable **(D)** between the two mouse strains. **(E)** OHCs from B6 mice had a more positive half-activation voltage (V_half_). N.S. means *p* > 0.05, ^**^ means *p* < 0.01 and ^***^ means *p* < 0.001.

While the vast majority of auditory signals is conveyed to auditory afferent fibers through IHCs, OHCs play a crucial role in amplifying sound-evoked vibrations and thus their impact on dictating hearing performance cannot be overstated. To assess the OHC function, we performed whole-cell patch-clamp recording and measured whole-cell membrane capacitance with two-sine protocol (Santos-Sacchi and Song, 2014). As previously demonstrated by one of our co-authors, we observed non-linear capacitance in OHCs from both mouse strains, which is directly related to their ability to amplify mechanical vibrations (Figure 5A). We then fitted capacitance-voltage relationship to the first derivative of the Boltzmann function, and we found that OHCs from B6 mice have more non-linear capacitance (Q_max_, 0.72 ± 0.05 vs. 0.86 ± 0.02 pC, for CBA and B6 mice, respectively, *n* = 7 and 5; unpaired Student's *i*-test, *p* < 0.001, Figure 5B) and more linear capacitance (C_lin_, 5.97 ± 0.68 vs. 7.69 ± 0.66 fF, *n* = 7 and 5; unpaired Student's *t*-test, *p* < 0.01, Figure 5C). However, when we calculated the ratio of Q_max_/C_lin_, we found no significant difference between the two mouse strains (0.12 ± 0.02 vs. 0.11 ± 0.01, *n* = 7 and 5; unpaired Student's *t*-test, *p* > 0.05, Figure 5D). Given that the specific membrane capacitance is relatively constant for different cell types (Gentet et al., 2000), C_lin_ is expected to be proportional to the total cell surface area, so that it can be taken as a measurement of cell size. Therefore, our results suggest that although OHCs from B6 mice are larger in size, the prestin density is similar between the two mouse strains. Finally, we observed that activating non-linear capacitance in OHCs from B6 mice required a more depolarized membrane potential (−48.5 ± 4.62 vs. −61.6 ± 3.78 mV, *n* = 7 and 5; unpaired Student's *t*-test, *p* < 0.001, Figure 5E), which may have a negative impact on their amplifying capability.

### Counting Ribbon Synapses and Spiral Ganglion Cells

**Figure 6:**
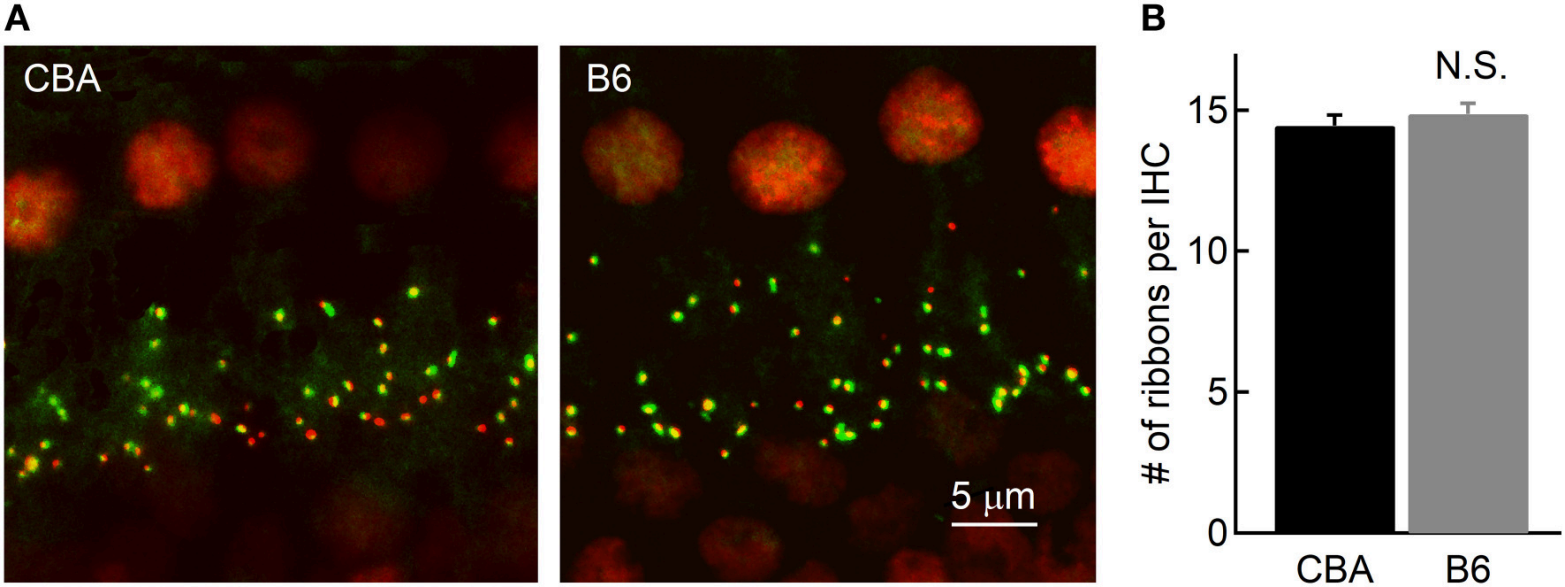
Counting the number of ribbon synapses in IHCs. **(A)** Confocal images of whole-mount immunostaining of two cochleae in the apical turn, which were double stained for CtBP2 (red) and GluR2 (green). **(B)** Ribbon synapses were counted based on fluorescence puncta, as shown in **(A)**. Note that the cochleae of the two mouse strains have a similar number of ribbon synapses per IHC.

It is puzzling that IHCs inB6 mice release more glutamate and thus function more efficiently in activating auditory afferent fibers, yet ABR Wave I amplitude in these mice is smaller (Figure 1D). To account for this discrepancy, we first wondered that if this is due to a smaller number of spiral ganglion cells (SGNs) contacting each IHC inB6 mice. We thus performed fluorescence staining on whole-mount organs of Corti of both mouse strains. We used two primary antibodies directed against Ribeye, a major structural component of synaptic ribbons (Schmitz et al., 2000), and post-synaptic AMPA receptor subunit 2 (GluR2). We defined each punctum of double staining as a ribbon synapse and we found no significant difference in the number of ribbon synapses per IHC between the two mouse strains (14.5 ± 1.79 vs. 14.9 ± 2.01, for CBA and B6 mice, respectively, *n* = 22 and 28; unpaired Student's *t*-test, *p* > 0.05; Figure 6), consistent with previous studies (Wang and Ren, 2012; Ohlemiller et al., 2016; Hickox et al., 2017; Cui et al., 2018). This result suggests the smaller ABR Wave I amplitude in B6 mice is not due to a smaller number of SGNs contacting each IHC.

**Figure 7:**
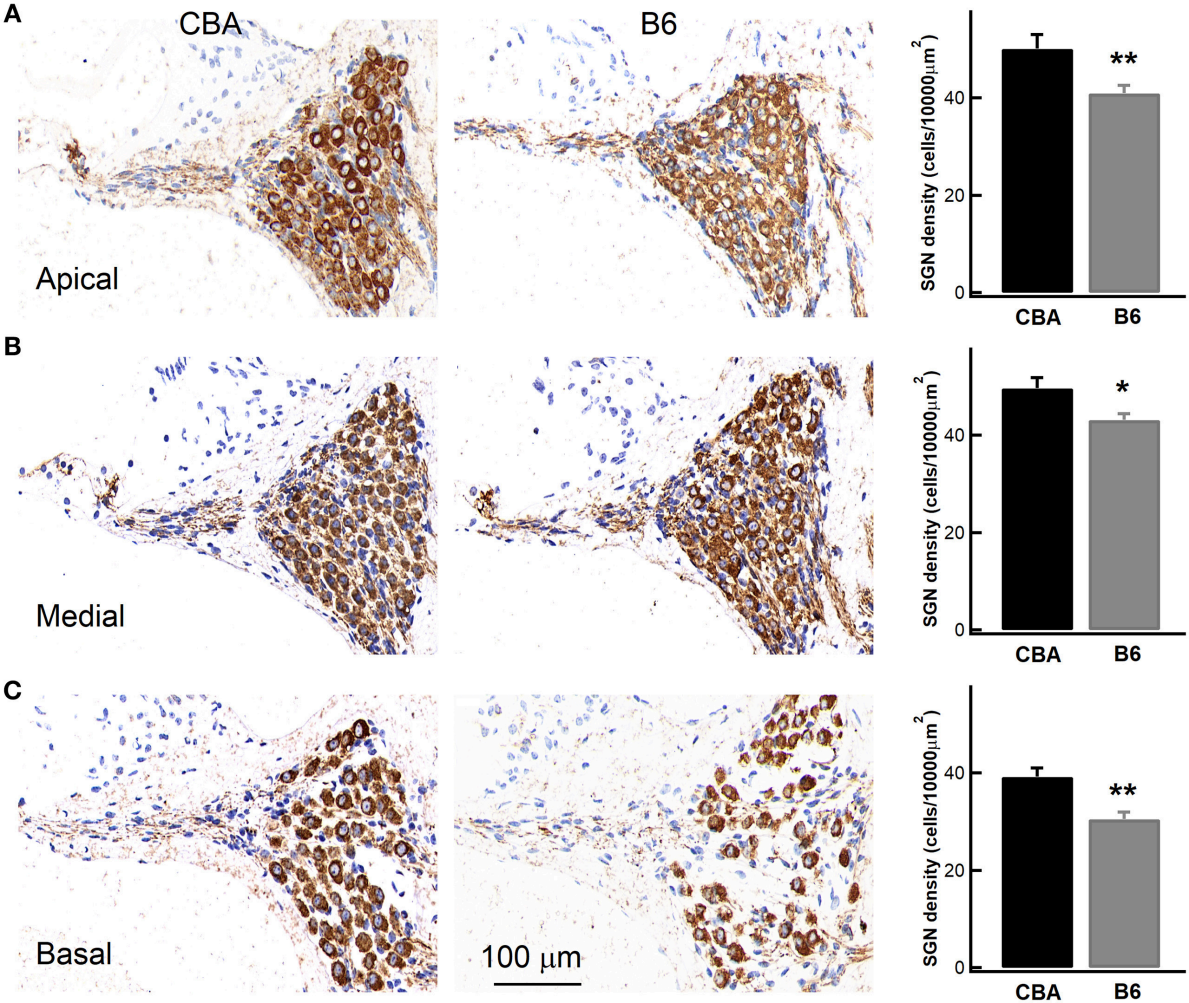
Examining the density of spiral ganglion neurons (SGNs) in the cochlea. Left, staining of cochlear sections in the apical **(A)**, medial **(B)**, and basal turn **(C)**. SGNs were stained with an antibody, which is depicted in brown. Right, SGN density, quantified as the number of SGNs per 10,000 μm^2^, was pooled from multiple experiments, showing that the B6 cochleae have significantly lower SGN density for all the three turns. ^*^ means *p* < 0.05 and ^**^ means *p* < 0.01.

Given that B6 mice have significantly larger OHCs (Figure 5C), we next wondered if they have fewer OHCs and IHCs, and therefore likely fewer SGNs. To count SGNs, we stained cochleae with TUJ1 (Figure 7) and examined cell bodies of SGNs found in cochlear modiolus. For quantitative comparison, we calculated the SGN density as the number of SGNs per 10,000 μm^2^ (Leake et al., 2011). In the apical turn, there are indeed fewer SGNs in B6 mouse cochlea (50.2 ± 8.92 vs. 40.9 ± 5.62, for CBA and B6 mice, respectively; *n* = 9 and 12, unpaired Student's *t*-test, *p* < 0.01, Figure 7). Similar results were observed for both medial (49.7 ± 6.68 vs. 43.2 ± 4.52, unpaired Student's *t*-test, *p* < 0.05) and basal turn (39.2 ± 5.52 vs. 30.5 ± 5.11, unpaired Student's *t*-test, *p* < 0.01). While we cannot rule out other possibilities, the smaller number of SGNs in B6 mice is likely to contribute to the smaller ABR Wave I amplitude in these animals.

## Discussion

Hearing loss is a complex disorder that can be attributed to many genetic and environmental factors, all of which are constantly interacting to shape the hearing performance. As two illustrating examples, CBA and B6 mice have been used extensively to explore mechanisms underlying hearing loss for many years (Li and Hultcrantz, 1994; Spongr et al., 1997; Brewton et al., 2016). B6 mouse strain is a long-lived strain and the most widely used mouse model for studying aging and age-associated diseases. Previous studies have shown that B6 mice carry a specific mutation in *Cdh23*, whose protein product binds with protocadherin 15 and forms tip links between adjacent stereocilia (Ohlemiller, 2006), and mutations in *Cdh23* lead to disorganized hair bundles (Noben-Trauth et al., 2003). Furthermore, it has been shown that hair cells with *Cdh23* mutations are more vulnerable to oxidative stress and more prone to apoptosis (Someya et al., 2009). In this present study, we sought to examine cellular differences in the cochlea of the two mouse strains in their juvenile stage, during which the hearing performance is comparable. We believe that these cellular differences are useful in interpreting current findings *in vivo*, especially those concerning early onset hearing loss and aging of hearing.

Hearing relies on faithful transmission of auditory signals from IHCs to SGNs across ribbon synapses in between (Wichmann and Moser, 2015). Voltage-gated Ca^2+^ channels lie beneath synaptic ribbons, and they are activated according to the graded membrane potential in IHCs and modulate exocytosis of synaptic vesicles. We found that Ca^2+^ current in IHCs from B6 mice has a larger peak amplitude and steeper voltage dependence and activates at a more negative membrane potential, all of which may function synergistically to bring substantially more Ca^2+^ influx into the cell under physiological conditions. This excessive load of Ca^2+^ into IHCs is likely to cause excessive exocytosis and glutamate toxicity, which has been directly linked to hearing loss. In addition, the excessive load of Ca^2+^ for a prolonged period of time could cause damages in IHCs by making them more prone to Ca^2+^-induced cytotoxicity (Verkhratsky and Toescu, 1998).

In response to Ca^2+^ influx, IHCs release glutamate-containing synaptic vesicles through exocytosis at their ribbon synapses. Previous studies have established that exocytosis from hair cells displays an initial quick phase, representing release of synaptic vesicles from the readily releasable pool (RRP), and a sustained release that could last for seconds, owing to the quick and efficient replenishment of synaptic vesicles (Johnson et al., 2005). We found that IHCs from B6 mice release more synaptic vesicles for both short and long step stimulations, suggesting that IHCs from these mice not only have a larger RRP but also replenish synaptic vesicles more quickly. Indeed, when we applied double-pulse stimulation, we found that exocytosis from B6 IHCs recovers more quickly, which is probably due to the greater Ca^2+^ influx (Babai et al., 2010). Interestingly, we found that Ca^2+^ is less efficient in triggering synaptic vesicle releases in IHCs from B6 mice. Owing to the greater Ca^2+^ influx, IHCs in B6 mice manage to release significantly more synaptic vesicles. This excessive release of synaptic vesicles could cause glutamate toxicity, which accumulates over time and eventually causes early onset hearing loss found in this strain of mice. However, while we would like suggest thatB6 mice are more prone to glutamate toxicity, it has been well documented that these mice show resistance to hidden hearing loss, a condition caused, at least in part, by glutamate toxicity (Shi et al., 2015a,b). It is likely that early onset hearing loss and hidden hearing loss are mediated through overlapping yet distinct mechanisms, and the involvement of glutamate toxicity in either condition remained to be carefully examined.

It is perplexing that although IHCs in B6 mice release more glutamate but the ABR Wave I amplitude is not any larger in these animals. On the contrary, it is indistinguishable from that of CBA mice for low SPLs, and it is even smaller when SPL increases beyond 70 dB. The ABR wave I amplitude represents sound-evoked spikes of all responding SGNs, and there is a strong correlation between the number of ribbon synapses and the ABR Wave I amplitude (Altschuler et al., 2015). Therefore, we examined both the number of ribbon synapses per IHC and the total number of SGNs in the apical turn in both mouse strains, and we found that while the number of ribbon synapses per IHC is similar between the two mouse strains, B6 mice have significantly fewer SGNs, which could account for the smaller ABR Wave I amplitude found in these animals (Lenzi et al., 2002; Pangrsic et al., 2015). Furthermore, we also found B6 mice have fewer SGNs at the medial and basal turn, which could contribute to the smaller ABR Wave I amplitude for louder sounds because louder sounds at 8 kHz are likely to activate SGNs in the medial and basal turn (Taberner and Liberman, 2005) and B6 mice have fewer SGNs available to be recruited across all frequencies.

The cochlear outer hair cells (OHCs) have been shown to be capable of amplifying sound-evoked vibrations (Santos-Sacchi and Dilger, 1988), owing to dense expression of the motor protein prestin in their lateral membrane. Therefore, changesin the prestin density in OHCs could have a significant impact on hearing sensitivity, which could be an underlying mechanism for hearing loss (Hoben et al., 2017). We thus examined the OHC function in both mouse strains by measuring linear and non-linear capacitance. We found that the prestin density, i.e., the ratio between linear and non-linear capacitance, is similar between the two mouse strains, consistent with the aforementioned finding that the two strains share similar hearing thresholds across all frequencies tested.

In summary, we found IHCs in B6 mice are more prone to Ca^2+^ overload and release excessive glutamate onto auditory afferent fibers, both of which could accumulate over a prolonged time of period and lead to substantial hearing loss. These findings provide useful clues for understanding hearing impairment in general and aging of hearing in particular.

## Data Availability

All datasets generated for this study are included in the manuscript and/or the supplementary files.

## Author Contributions

HW and G-LL designed and supervised the whole study. LS designed the experiments in outer hair cells and GL carried out the experiments. HL, JL, and Y-GG performed the rest of experiments and wrote the manuscript.

### Conflict of Interest Statement

The authors declare that the research was conducted in the absence of any commercial or financial relationships that could be construed as a potential conflict of interest.
